# Analysis of Mercury in Aquifers in Gold Mining Areas in the Ecuadorian Amazon and Its Associated Risk for Human Health

**DOI:** 10.3390/toxics12020162

**Published:** 2024-02-19

**Authors:** Irene Passarelli, Michelle Vanessa Villacis Verdesoto, Samantha Jiménez-Oyola, Ana Gabriela Flores Huilcapi, Demmy Mora-Silva, Giorgio Anfuso, Jose Fernando Esparza Parra, Mirian Jimenez-Gutierrez, Luis Santiago Carrera Almendáriz, Victor Gabriel Avalos Peñafiel, Salvatore Straface, Carlos Mestanza-Ramón

**Affiliations:** 1 Department of Environmental Engineering, University of Calabria, 87036 Rende, Italy; pssrni88t62i854p@studenti.unical.it (I.P.);; 2 Green Amazon, Research Center, Nueva Loja 210150, Ecuador; mivavill@espol.edu.ec (M.V.V.V.); luissantiago.carrera@espoch.edu.ec (L.S.C.A.);; 3 Escuela Superior Politécnica del Litoral, ESPOL, Facultad de Ingeniería en Ciencias de la Tierra, ESPOL Polytechnic University, Campus Gustavo Galindo km 30.5 Vía Perimetral, P.O. Box 09-01-5863, Guayaquil 090101, Ecuador; 4 Faculty of Food Science and Engineering and Biotechnology, Universidad Técnica de Ambato, Av. Colombia y Chile, Ambato 180104, Ecuador; 5 Research Group YASUNI-SDC, Escuela Superior Politécnica de Chimborazo, Sede Orellana, El Coca 220001, Ecuador; 6 Department of Earth Sciences, Faculty of Marine and Environmental Sciences, University of Cádiz, 11510 Puerto Real, Spain; giorgio.anfuso@uca.es; 7 Escuela Superior Politécnica de Chimborazo (ESPOCH), Faculty of Natural Resources, Panamericana Sur km 1 ½, Riobamba 060155, Ecuador

**Keywords:** artisanal small-scale mining, groundwater pollution, environmental and health risks, Ecuadorian Amazon, risk evaluation

## Abstract

Gold mining activity is a source of supply in many areas of the world, and especially in developing countries, it is practiced illegally and by applying unsafe techniques. Particularly in Ecuador, artisanal and small-scale gold mining (ASGM) is widespread, and it is based on the use of toxic substances, such as mercury (Hg), in gold recovery. Hg is a heavy metal that is water-insoluble, which, once mobilized, poses a threat to both the environment and human health. This study analyzes Hg concentrations in the six provinces of Napo, Sucumbíos, Orellana, Pastaza, Morona Santiago, and Zamora Chinchipe of the Ecuadorian Amazon region to conduct a human health risk assessment. Significant differences in Hg levels were found between provinces, but concentrations were below MPL imposed by Ecuadorian regulations everywhere. Nevertheless, a worrisome picture emerges, especially with regard to the most vulnerable receptors represented by the child population. There are multiple factors of incidence that may affect the possible future development of the phenomenon, and with reference to the social, economic, and environmental context of the region, it can be concluded that it may be appropriate to plan further investigation to arrive at a more comprehensive assessment. The results of this study can be used by decision makers to plan further investigation and to implement monitoring networks, risk mitigation strategies, and groundwater protection measures.

## 1. Introduction

The present investigations were conducted to determine the concentration of mercury (Hg) in the coastal region of Ecuador to assess the resulting risks to the environment and human health using both deterministic and probabilistic methods.

Artisanal and small-scale gold mining (ASGM) is an important source of economic livelihood in many areas of the world (where mineral deposits exist), especially in rural areas where employment opportunities are very limited [1]. In these areas, in most cases, this activity is carried out illegally and in violation of present environmental regulations. The most serious associated problem is the extensive use of mercury (Hg) in gold recovery processes [2]. In recent decades, the problem has worsened as global demand for gold has increased, driving criminal groups to finance illegal gold mining and, to increase production, processes have shifted from rudimentary to mechanized, resulting in even greater use of Hg [3]. In Ecuador, mining can be described as a historical activity dating back to colonial and precolonial times. However, in the 1990s, it increased significantly when the existence of precious metal deposits was established through geological studies financed by the Central Bank [4]. In its beginnings, mining was carried out with rudimentary techniques; in the last decades, an important enhancement of the methods used, which vary from place to place, has been recorded. It can be carried out at different scales and in different ways; it often continues to be carried out illegally or informally, and in some cases, local authorities tend to tolerate it [5]. Currently, medium- and large-scale mining projects have developed and consolidated alongside artisanal and small-scale activities [6].

Hg is considered one of the most hazardous heavy metals for both human health and the environment [7]. It can enter the atmosphere as inorganic mercury from geogenic and anthropogenic sources and then be converted to organic mercury by natural processes. The organic forms of Hg mercury, such as methylmercury and dimethylmercury, are the most toxic and harmful [8,9]. The effects of Hg on human health can vary depending on the duration of exposure and the level of toxicity. The toxicity of a metal is influenced by several factors: chemical form, rate of absorption and elimination from an organism, and tissue concentration [10]. The main effects of short-term exposure include nervous system abnormalities and disorders of the respiratory and gastrointestinal tract [5,11]. In cases where exposure is more prolonged or toxicity levels are higher, the effects can be much more severe. These include cancer, DNA mutations, cardiovascular system dysfunction, and damage to the central nervous system, blood cells, and vital organs such as the lungs, liver, and kidneys [10,12].

There are multiple migration pathways by which a contaminant can reach human receptors, but in general, these depend on the particular environment under consideration and the species prevalent there [13]. For residential and recreational uses, the most frequent mode of exposure appears to be accidental ingestion of contaminated water. Added to this is the consumption of fish caught near mining areas, which can introduce organic mercury (methylmercury) into the human body [13,14]. In workplaces where mining is improperly conducted and where amalgamation processes involving mercury are carried out, inhalation of elemental Hg vapors is the most frequent type of exposure [15,16]. A final mode of exposure that deserves mention is dermal contact. This phenomenon is not simply a contaminant but plays a crucial role in exposure to harmful substances [17]. According to this scenario, people living near areas where mining is practiced are subject to numerous exposure pathways. Therefore, they have a high probability of developing adverse health effects.

In the Amazon region of Ecuador, mining has traditionally been carried out by applying rudimentary techniques based on the use of Hg in amalgamation processes, although this has been banned since 2015 [18]. These methods were widely used, especially in the southern region, i.e., in the jurisdiction of Chinapintza (Zamora Chinchipe province). In this region, the impacts of Hg use and inadequate management of tailing dumps have mainly resulted in deforestation and water contamination. Several studies have shown a significant increase in gold mining activities in four provinces of the Ecuadorian Amazon: Napo, Orellana, Sucumbíos, and Zamora [19,20,21,22].

A large part of the Amazonian population lives from activities such as agriculture, so mining also emerges as a key and attractive activity. Recent studies have found an alarming expansion of illegal mining in the provinces of Napo, Orellana, Sucumbíos, Pastaza, Morona Santiago, and Zamora Chinchipe, with evident effects of deforestation and contamination of surface and subsurface waters [23,24]. The present investigation is focused on determining mercury concentrations in groundwater near mining areas and assessing the resulting risk to the human health of exposed populations using deterministic and probabilistic methods. To carry out the risk assessment, 147 water samples were taken from wells near the mining areas. The deterministic approach performs a point-type analysis, while the probabilistic approach is based on modeling a probability distribution. This will allow the spatial pattern of social and environmental effects to be determined to fully understand and better manage the risk, providing decision makers with a tool to implement the pollution mitigation strategy.

The aim of this study is to examine Hg concentrations in groundwater in the Ecuadorian Amazon and assess the corresponding health risk. To date, there are no further studies on groundwater in the Amazon region. There are interactions between different environmental compartments, so groundwater contaminants can easily reach receptors. The guardianship of groundwater is extremely important for the protection of human health, considering that it accounts for almost all drinking water resources. In addition, it feeds the spring and, especially in lean periods, the flow of surface water courses. Therefore, water contamination can quickly spread to humans, the fish population, and vegetation. In the case of Hg, the situation is even more worrying, considering that this metal is water-insoluble. The results of this study can be used as a guideline for planning more detailed investigation plans, risk mitigation actions, and groundwater protection interventions.

## 2. Materials and Methods

### 2.1. Study Area

**Figure 1:**
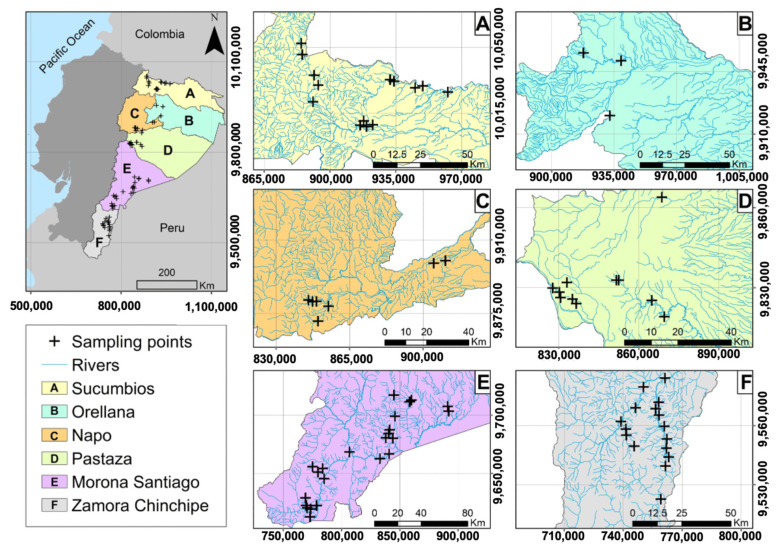
Study area and location of the sampled sites.

The study covers six provinces in the Amazon region of Ecuador: Sucumbíos, Orellana, Napo, Pastaza, Morona Santiago, and Zamora Chinchipe (Figure 1). This area includes six watersheds and a population of 739,814 inhabitants [25]. This area has been characterized mainly by economic activities such as cattle ranching, agriculture, and, since the end of the 1960s, oil and gold mining activities. These last two activities have intensified in recent decades, causing serious impacts to the biotic, abiotic, and social environment. These impacts are directly related to the release of heavy metals and other pollutants residues from mining processes [13]. In the Amazon Region, there are 225 mining concessions, of which two are of the general regime, 164 correspond to small mining, five to medium, and 54 to large mining, of which 50 are in the exploitation stage, mainly in the south of the region in the provinces of Morona Santiago and Zamora Chinchipe. In addition, in the last ten years, the region has seen a notable increase in socio-environmental conflicts caused by illegal gold mining. Moreover, the impacts caused by gold mining activity have significantly affected surface water bodies due to the indiscriminate discharge of mining waste and the use of Hg in amalgamation processes [21,26].

### 2.2. Sampling and Laboratory Analisys

The field work was carried out between March and July 2022, a period characterized by rainfall between 200 and 350 mm [27]. Samples were collected from domestic water consumption wells in hamlets or rural communities located near the mining concessions (Figure 1). This study focused on analyzing the concentration of Hg in water samples from aquifers. Regarding the procedure for taking and transporting the samples, 250 mL amber flasks were used and subjected to acidification with 0.10 mL of nitric acid. All the samples were transported under a rigorous chain of custody to the Science Laboratory of the Escuela Superior Politécnica de Chimborazo, located in Orellana, Ecuador. The procedure to determine the Hg concentration was based on atomic absorption and hydride generation (atomic absorption spectrophotometry; PG Instruments, Leicestershire, UK). The application of the method for the determination of mercury (Hg) concentration has been preceded by a procedure for quality assurance and quality control protocol in accordance with the EPA method 7473 [25]. Analyses were conducted by operators wearing safety equipment to avoid any potential contact (ingestion, inhalation, and dermal absorption) with mercury (Hg) compounds. In addition, a quality check was carried out on water samples to detect samples with a high organic content. Such samples require special care during analysis, and the sample size should be reduced to avoid ignition of the samples in the decomposition tube.

First of all, the laboratory demonstrated its initial proficiency by following the sample preparations and analysis procedures according to the method. For each batch of samples processed, both a blank method and a laboratory control sample were carried out. A blank method is prepared by using a reagent of water at the specified volume and carried through the appropriate steps. If the method blank does not contain the target analyte at a level that interferes with the quality of the project, it is considered acceptable. In the absence of specific indications, this means that a maximum level of 10% of the lowest concentration of the sample is considered acceptable. The laboratory control samples must be carried out for each analyte of interest at the project level. Acceptance criteria can be set based on historical data, or in the absence of these, the limit can be set at ±20% of the spiked value.

The Hg measurement range was from 0.0005 to 10 mg/L, and the reference method used was Standard Methods, Ed. 23. 2017, 3112B-Acid Digestion: EPA Method 3015, 2007. Samples were prepared according to the nitric acid digestion procedure described in EPA Method 7473 [28]. The entire process, from sample collection to processing in the laboratory, complied with international quality, confidentiality, and code of ethics policies.

#### 2.2.1. Brief Description of EPA Standard Method 3015

An aqueous sample undergoes extraction with concentrated nitric acid or a combination of concentrated nitric acid and hydrochloric acid, using microwave heating in a suitable oven. The acidified sample is then placed in a microwave-friendly container made of fluorocarbon polymer (DuPont, Wilmington, DE, USA) (such as PFA or TFM) or quartz or placed in a container liner. The container is closed and heated in the microwave oven for a time. After cooling, the contents of the container are filtered, centrifuged, or allowed to settle, then diluted to volume and ready for analysis (Environmental Protection Agency).

#### 2.2.2. Brief Description of EPA Standard Method 7473

EPA Method 7473 is a suitable standard for the analysis of mercury (in organic and inorganic forms) in sediment and aqueous samples or in digested solutions. Application of this method requires prior full calibration of the instrument’s working range. This is performed at the initial stage by setting the significant instrumental parameters and is called primary calibration. The primary calibration should be performed after the replacement of the decomposition tube, amalgamator, or oxygen tank. This is followed by a daily calibration to ensure that the primary calibration is valid. The products and instruments used are in accordance with the indications of this standard; thus, the suitability of performance has been documented for them (Environmental Protection Agency).

Controlled heating in an oxygen-containing decomposition oven releases mercury from solid and aqueous samples into the instrument. The sample is dried and then thermally and chemically decomposed in the decomposition oven. The decomposition products are transported to the catalytic part of the furnace by a stream of oxygen. The oxidation is complete, and halogens and nitrogen/sulfur oxides are captured. The remaining decomposition products are transported to an amalgamator, which selectively captures the mercury. After the system is oxygenated to remove any remaining gases or decomposition products, the amalgamator heats up rapidly and releases mercury vapors. The oxygen stream transports mercury vapor through absorption cells located in the light path of a single-wavelength atomic absorption spectrophotometer (Thermo Fisher Scientific, Waltham, MA, USA). The absorbance (peak height or peak area) is measured at 253.7 nm as a function of mercury concentration (Environmental Protection Agency).

### 2.3. Risk Assessment and Characterization

For the human health risk assessment, the residential scenario was considered. The main routes of exposure were water ingestion and dermal contact. The potential risk associated with human health was assessed by the hazard quotient (HQ) calculated for non-carcinogenic type substances, defined as the ratio between the average daily dose (ADD) and the reference dose (RfD). The average daily dose (ADD) was determined by Equations (1) and (2), USEPA 2001 and 2004, respectively. The RfD-Hg was obtained from the Risk Assessment Information System website. The risk was evaluated in terms of cumulative risk through the parameter known as the hazard index (HI), given by the sum of the HQs related to the two exposure routes. If HQs or HI have values above 1, it means that the safe exposure threshold has been exceeded and, therefore, adverse effects related to Hg exposure may occur. Both the traditional deterministic method and the probabilistic method were applied to perform the risk assessment.

(1) $${ADD}_{ingestion}=\frac{C_{sw}\cdot EF\cdot IR\cdot ED}{AT\cdot BW\cdot CF}$$

Equation (1)—Average daily dose by the route of ingestion, USEPA 2001.

(2) $${ADD}_{dermal\;contact}=\frac{C_{sw}\cdot EF\cdot ET\cdot ED\cdot SA\cdot k_{p}}{AT\cdot BW\cdot CF}$$

Equation (2)—Average daily dose by the route of dermal contact, USEPA 2004, where the meaning of the parameters is as follows:

$$\left\{\begin{array}{c} C_{sw}=Hg\;concentration\;in\;water\;(\frac{mg}{L})\\ EF=exposure\;time\;(\frac{days}{year})\\ IR=ingestion\;rate\;of\;water\;\left(\frac{L}{day}\right)\\ ET=exposure\;time\;\left(\frac{hours}{event}\right)\\ ED=lifetime\;exposure\;duration\;\left(years\right)\\ SA=skin\;surface\;area\;exposed\;\left({cm}^{2}\right)\\ k_{p}=skin\;permeability\;constant\;\left(\frac{cm}{hour}\right)\\ AT=averaging\;time\;\left(days\right)\\ BW=body\;weight\;\left(kg\right)\\ CF=conversion\;factor \end{array}\right.$$

Insight into the significance of parameters:

- C_SW_ (Concentration): It represents the concentration of mercury detected at selected sampling points.
- EF (Exposure Frequency): It is a representative parameter of the average number of days per year the receptor is considered to be exposed to contamination.
- IR (Ingestion Rate): It represents, on average, the amount of contaminated water ingested daily by the receptor. Clearly, this quantity varies depending on the type of receptor.
- ET (Exposure Time): It represents the duration of exposure with reference to the individual contamination event.
- ED (Exposure Duration): It represents the duration, expressed in years, over which, on average, the receptor is considered to be exposed to contamination. Therefore, the ED value varies depending on the type of receptor: adult or child.
- SA (Skin Area): It is the average area of skin considered to be exposed to contamination through dermal contact. It varies depending on the type of receptor considered.
- Kp (Skin Permeability Constant): The amount of contaminant absorbed per centimeter of skin exposed per hour.
- AT (Averaging Time): It represents the period over which the exposure is averaged.
- BW (Body Weight): It is the average body weight of the receptor, so it has a different value for adult and child receptors.
- CF (Conversion Factor): It is used to standardize units of measurement.
- RfD (Chronic Reference Dose): It represents the maximum dose of toxic contaminant that can be accepted. In essence, it is the concentration value of the pollutant for which no adverse effects on human health have been found in the literature.

Regarding the values to be used for each parameter, depending on the receptor type, the values recommended by international standards are considered. Table 1 presents the values assigned to each parameter and the probability distributions used in the human risk assessment. 

## 3. Results

### 3.1. Hg Concentration in Water

The summary statistics of Hg concentration in the groundwater samples for each province are presented in Table 2. The Hg concentration for the 50th percentile (p50) decreased in the following order: Napo > Sucumbíos > Morona Santiago > Pastaza > Zamora Chinchipe > Orellana. Forty-one percent of the samples were below the detection limit of the measuring equipment (LoD = 0.0005 mg/L). This could indicate that the concentrations are below the instrument’s detection threshold. However, undetected values could also be caused by malfunctions in the instrumentation or accidental errors on the part of the operators. In such cases, it may be appropriate to repeat the measurements several times. Moreover, it is possible to consider constructing a new data set cleaned of non-detected values. In the present study, a Hg concentration of half the MPL was assumed at the points where the values were not detected.

With the exception of Orellana province, generally, the Hg concentration was in the range of 0.0007 mg/L and 0.0056 mg/L; therefore, 100% of the samples presented Hg concentration below the MPL established in the Ecuadorian regulation for water quality for human consumption (0.006 mg/L) (INEN 1108).

As a background, it should be noted that there is currently no information about the mercury concentration in groundwater in the Amazon region of Ecuador. Studies to date have reported Hg concentration in surface water, sediments [13,20], soils [21], biological samples [33], and fish [34]. Thus, our study constitutes a baseline for future research concerning groundwater quality in the region.

The Hg concentration in groundwater detected in our study (0.0007 and 0.0056 mg/L) was in the range of Hg values reported in surface waters in the Ecuadorian Amazon; Mestanza-Ramón et al. [13] reported Hg concentrations between 0.0005 and 0.0070 mg/L in several rivers and streams of the Amazon region; and Capparelli et al. [20] reported Hg concentrations between 0.0005 and 0.0112 mg/L in 14 tributaries of the Napo River, however, the observations only partially match these values. This relationship between Hg concentrations in surface and groundwater is evidence of the interaction between these two environments and may be related to infiltration processes that occur when Hg present in surface water bodies is mobilized to the aquifer [35]. These findings highlight the importance of determining the sources of aquifer recharge in order to identify potential sources of contamination that may be affecting the quality of groundwater resources [36].

Several studies have reported the presence of various anthropogenic sources of contamination in the Ecuadorian Amazon, which can affect water quality, both surface and groundwater; among the main ones are artisanal and small-scale gold mining (mainly carried out illegally), inadequate management of mining environmental liabilities [5], fish farming [20], and natural Hg leaching from deforestation [37]. Therefore, assessing water quality in these environments is essential to ensure the protection of water resources and, thus, the safety of users.

Additionally, the results of our study were compared with studies that evaluated Hg concentration in groundwater worldwide. Ghana [38] reported Hg values between 0.00001 mg/L and 0.0132 mg/L in well water samples in areas with auriferous mining influence. In Saudi Arabia [39], the presence of Hg in groundwater in areas near the Mah Adh Dhahab gold mine, where Hg concentrations of up to 9.990 mg/L were recorded, was evaluated. In Nigeria, ref. [40] analyzed Hg concentrations and detected average concentrations of 0.0001 mg/L. In this context, the Hg concentrations detected in the Ecuadorian Amazon are within the range of the values reported in the aforementioned studies but tend to be lower than the maximum values detected in Saudi Arabia. Despite this, it is important to note that Hg levels measured in the Ecuadorian Amazon may represent a significant long-term risk to both health and the environment and should, therefore, be monitored regularly.

### 3.2. Human Health Risk Assessment

#### 3.2.1. Deterministic Approach

**Figure 2:**
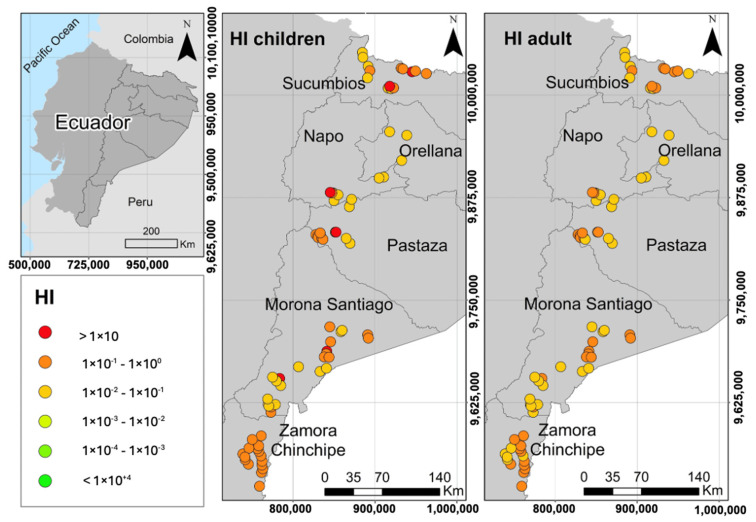
Point risk map of hazard index (HI) for receptors exposed to polluted ground waters in the Ecuadorian Amazon.

The point risk maps obtained from the deterministic assessment are presented in Figure 2. In addition, Table 2 summarizes the deterministic non-carcinogenic risk values (HI) for the 50th percentile (p50) of Hg exposure for each province assessed and for each receptor. HI values > 1 were reported in the provinces of Sucumbíos, Napo, Pastaza, and Morona Santiago. As shown in Figure 2, six of the sampled sites are above the acceptable risk threshold (HI > 1) for minor receptors, which are the most vulnerable to Hg exposure.

The HQ values for water ingestion were below 1 for adults (in the order of 10^−1^ and 10^−2^) (HI < 1) but above 1 in children in four of the six provinces analyzed; in Sucumbíos, two sites were recorded with HI values slightly above the safe exposure threshold; in Napo and Pastaza, a single sampling point was recorded where the HI was greater than 1; and in Morona Santiago, two sites were observed that represent risk of exposure. Morona Santiago had the highest non-carcinogenic risk value in children (HI = 1.49). In contrast, in Orellana and Zamora Chinchipe, the systemic risk values do not exceed the safe exposure limit for both receptors.

The results of our investigation agree with the findings reported by Mestanza-Ramón et al. [13], who evaluated the systemic health risk from exposure to surface water in the Amazon region through ingestion, obtaining deterministic risk values (p95) in children of HI = 2.51 in Sucumbíos, HI = 2.34 in Orellana, HI = 2.56 in Napo, HI = 2.90 × 10^−1^ in Pastaza, HI = 8.80 × 10^−1^ in Morona, and HI = 1.58 in Zamora Chinchipe. As expected, the risk values for surface water exposure, mainly in children, were significantly higher than those reported for groundwater exposure. Likewise, Jiménez-Oyola et al. [29] evaluated the deterministic risk to human health from exposure to surface water for recreational purposes in the province of Napo, obtaining values that exceeded the safe exposure threshold, with an HI = 1.85 for adults and an HI = 4.83 for children, values significantly higher than those determined for exposure to groundwater in our study.

The most relevant route of exposure with the highest contribution to risk was water ingestion, with HI values between 2.26 × 10^−2^ and 5.07 × 10^−1^ for adults and in the range of 6.56 × 10^−2^ to 1.47 × 10^1^ for children. Regarding the dermal contact route, this presented very low HQ values, in the range of 8.02 × 10^−4^ and 1.80 × 10^−2^ for adults and between 1.17 × 10^−3^ and 2.63 × 10^−2^ for children; for both cases, the risk from dermal contact was below the acceptable limit for the receptors, so it is considered that this route of exposure presents a negligible contribution in the evaluation of the total risk. Similar results were reported by Mestanza et al. and Jiménez-Oyola et al. [13,29] in risk studies conducted in the Ecuadorian Amazon, with ingestion being the route that contributes most to the entry of Hg into the human body, according to the risk scenarios evaluated in the aforementioned studies. Despite this, it is important to consider other routes of exposure, such as inhalation of Hg in sites where illegal mining is carried out and Hg is used in amalgamation processes, and the risk assessment of food consumption should also be considered, as these can bioaccumulate Hg in their tissues. All these exposure scenarios can increase the risk to which the residents of potentially contaminated areas are exposed. Hence, a comprehensive risk study is essential to protect the health of the communities.

The impact of Hg on human health is widely known. The consumption of Hg-contaminated groundwater poses a risk to living beings, even in minute concentrations [41]. For example, in Pakistan, ref. [42] showed that the groups most affected by Hg exposure were young children and pregnant women using well water for domestic and agricultural purposes. Even during gestation, Hg in its organic form can cross the placental barrier between mother and fetus, causing brain damage and mental retardation [43]. For this reason, the World Health Organization (WHO) [44] recommends a maximum value of 0.006 mg/L Hg for drinking water consumption; Hg values above this limit can cause kidney damage, especially in children through long-term ingestion [45]. On this basis, the wells sampled in this study, despite having Hg concentrations slightly below the limit established by WHO, are not extensive enough to cause long-term health risks to the child population, so periodic sampling of groundwater quality is recommended, in addition to taking biological samples to assess the presence of Hg in the most vulnerable populations.

#### 3.2.2. Probabilistic Approach

**Figure 3:**
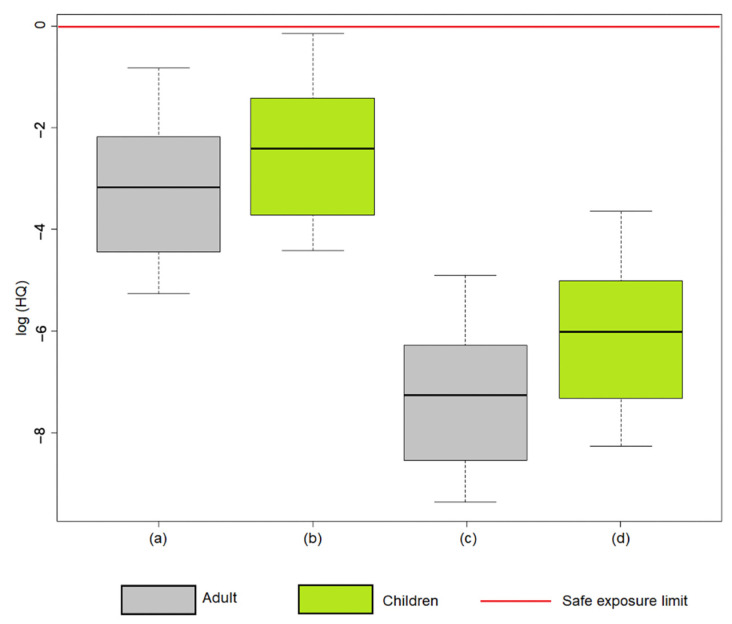
Boxplot of hazard quotient for (**a**) adult ingestion, (**b**) child ingestion, (**c**) adult dermal contact, and (**d**) child dermal contact. HQ values are given in logarithm.

The results of the probabilistic risk analysis yielded values below the safe exposure limit for adults and children; thus, according to the probabilistic analysis, there are no exposure levels that require attention for the receptors (Figure 3 and Table 3). The hazard quotient (HQ) values for children ranged between 1.2 × 10^−2^ and 8.66 × 10^−1^ for water ingestion and between 2.65 × 10^−4^ and 2.71 × 10^−2^ for dermal contact. For adults, the risk was lower, in the range of 5.14 × 10^−3^ and 4.39 × 10^−1^ for ingestion and 8.55 × 10^−5^ and 7.42 × 10^−3^ for dermal contact. In this sense, it is evident that, although the quantified risk for children is higher than the risk detected in adults, in both cases, the safe exposure limit is not exceeded.

The results of the deterministic analysis differ from the results of the probabilistic analysis (Table 4); according to the probabilistic methodology, the residential scenario does not generate risk for adult and child receptors, whereas, according to the deterministic methodology, there are sites where exposure generates risk for minor receptors. Therefore, based on a conservative criterion, the use of groundwater for domestic consumption is not recommended, mainly in sites located in areas of mining activity that show signs of contamination, since prolonged exposure of vulnerable populations to Hg could affect their health.

To have more conclusive results on the risks for the population, a detailed investigation should be carried out that includes in situ data on exposure and more Hg determinations in different matrices (soil, sediments, surface water, food, human tissue, etc.), with the aim of making a comprehensive risk assessment and minimizing the uncertainty in the risk assessment. It is also recommended to analyze other potentially toxic elements since other contaminants may be present in illegal mining operations that could affect the environment and the population.

## 4. Discussion

The results of the study carried out show a worrying scenario regarding Hg concentrations in the Amazon region of Ecuador. The samples to be analyzed were collected from groundwater bodies close to gold mining activities, so some provinces, being more affected, have a denser network of measurements, such as Morona Santiago, Zamora Chinchipe, and Sucumbíos. It can be concluded that the most affected areas in the region are the south and the north, where the average concentration obtained is between 0.0007 mg/L and 0.0056 mg/L. This value is below the maximum permissible limit imposed by Ecuadorian regulations for water intended for human consumption. However, the real risk to the population also depends on the vulnerability of the exposed receptors. In fact, the analysis showed that the child population is at greater risk.

To gain a more objective overview, the measured concentrations were also compared with the maximum permissible limits imposed by regulations in other countries, again with regard to water intended for human consumption. In particular, with respect to the European environmental quality standard (EQS) for groundwater bodies, all analyzed samples exceeded the maximum admissible limit of 0.07 μg/L. By contrast, with reference to the Canadian water quality guidelines, about 35% of the samples exceeded the maximum permissible limit of 0.001 mg/L. Therefore, although not exceeding legal limits, the detected Hg concentrations must be interpreted as a wake-up call. Firstly, it may be necessary to conduct more in-depth analyses in certain areas. Furthermore, it would be advisable to keep these areas under constant observation to ensure that Hg levels do not rise further.

According to many experts in the field, the high levels of Hg found in the analyzed samples can be directly attributed to gold mining, especially illegal gold mining, which is conducted with disregard for both environmental safety and the safety of the workers themselves [5,20,46]. The effects on the environment are manifold and, among others, include deforestation and contamination of groundwater bodies [5,22]. When a contaminant is released into the environment, it interacts with the environment and, depending on its chemical and physical properties, can move from one environmental compartment to another [47]. This increases the likelihood that the contaminant itself will spread and come into contact with human receptors. Thus, from the deep soil, the contaminant can reach the groundwater, flow back up to the soil, and contaminate surface water bodies and vice versa. Moreover, polluted water poisons fish fauna [48]. In addition, it can be used in agriculture for irrigation and contaminate agricultural products for human consumption. Finally, contaminated water can come into direct contact with humans through the known exposure routes of ingestion and dermal contact. The growth in Hg use in Ecuador has been proportional to the increase in gold mining activities [49]. Although the state has sanction instruments at its disposal, it has not yet used them to counter this problem [10,44]. This is partly due to the difficulty in controlling all the illegal activities taking place in the territory and partly due to inefficiency on the part of the administrations. Similar situations are widespread in other developing countries, such as Venezuela [50], Brazil [51,52], Colombia [53,54], and Peru [7]. In these countries, the indiscriminate use of mercury has caused severe impacts on the environment and human health [55]. To reverse the trend, the state of Ecuador should first increase control and monitoring activities in order to unmask illegal gold mining activities. Secondly, the state could create fiscal instruments to foster the creation and growth of mining activities that work within the legal framework. In this way, on the one hand, the state would alleviate the current problem of contamination by Hg, and on the other hand, it would favor the economic growth of the country [56].

## 5. Conclusions

The present study detected Hg concentrations in groundwater in the Amazon region of Ecuador. The study conducted found that the most affected provinces are Napo, Sucumbíos, and Morona Santiago. In contrast, in the province of Orellana, all samples analyzed exhibited Hg concentrations below the detection threshold of the measuring instruments used. Overall, the totality of samples analyzed (100%) had Hg concentrations below the MPL established by Ecuadorian regulations for water for human consumption. Although below legal limits, Hg levels detected in some provinces are significantly high. In addition, taking into account the fact that the mining activity is constantly expanding, the Hg level may increase at a later date. The overall picture suggests that the most affected provinces should be subjected to more control and possibly constant monitoring.

In any case, the results of the risk analysis vary depending on the two approaches used: the analysis carried out with a deterministic approach suggests a more severe scenario. This is because the deterministic approach is based on point-by-point analysis and, therefore, requires a significant amount of data. Instead, the probabilistic approach is based on the synthetic generation of a larger sample of data. This allows statistical uncertainties to be taken into account and provides a more complete view.

Nevertheless, a more accurate risk analysis is required to reconstruct a model that takes into account the following aspects: social, ecological, and geological. In fact, the risk is proportional to the actual vulnerability of the receptors, which in turn is influenced by several factors, such as the uses and habits of the exposed populations, the properties of the environmental compartments, and the geological and geomorphological properties of the soil. Knowledge of the properties of environmental compartments provides information on the essential interactions between them and the pollutant. Similarly, the geological characteristics of the subsurface provide a better understanding of the transport mechanism of the pollutant that may develop. Finally, knowing the social habits of the exposed population makes it possible to trace the most likely routes of exposure. The results obtained from this study can be used, together with other research, to plan monitoring and risk mitigation activities resulting from the use of Hg.

## Figures and Tables

**Table 1:** Parameters used in the risk assessment.

	Deterministic	Probabilistic	
Parameters	Value	Distribution	Values
EF	350	Triangular	(26–260)
ED (adults)	120	LogNormal	11.36 ± 13.72
ED (children)	0.22	Uniform	(1–6)
ET (adults and children)	2.6	Triangular	(0.5–6)
SA (adults)	23,000	Normal	18,400 ± 2300
SA (children)	7380	Normal	6800 ± 600
BW (adults)	72	Normal	72 ± 15.9
BW (children)	15.6	Normal	15.6 ± 3.7
IR (adults)	2.04	-	2.04
IR (children)	1.28	-	1.28
Kp	0.001	-	0.001

EF = Exposure Frequency; ED = Exposure Duration; ET = Exposure Time; SA = Skin Area; BW = Body Weight; IR = Ingestion rate; Kp = Skin Permeability Constant [29,30,31,32].

**Table 2:** Hg concentration (mg/L) in groundwater samples.

Province	*n*	Min–Max	p50	S.D.
Sucumbíos	14	0.0009–0.0044	0.0030	0.0013
Orellana	3	*	*	*
Napo	7	0.0029–0.0045	0.0037	0.0011
Pastaza	12	0.0009–0.0045	0.0022	0.0013
Morona Santiago	23	0.0008–0.0056	0.0026	0.0015
Zamora Chinchipe	16	0.0007–0.0037	0.0015	0.0010

*n* = number of samples; min = minimum; max = maximum; p50 = percentile 50; S.D. = Standard Deviation. * Values below the detection limit of the measuring equipment (LoD = 0.0005 mg/L) 0.006 mg/L = limit of Hg for human health protection in drinking water (INEN 1108). In this case, a value of half the LoD (0.00025 mg/L) is assumed for the purpose of the analysis.

**Table 3:** Deterministic HI from exposure to Hg in groundwaters for both receptors by province.

Province	Adults				Children			
	Min–Max	p50	p95	S.D.	Min–Max	p50	p95	S.D.
Sucumbíos	2.34 × 10^−2^–4.13 × 10^−1^	9.85 × 10^−2^	4.07 × 10^−1^	1.53 × 10^−1^	6.67 × 10^−2^–1.17	2.80 × 10^−1^	1.16	4.36 × 10^−1^
Orellana	2.34 × 10^−2^–2.34 × 10^−2^	2.34 × 10^−2^	2.34 × 10^−2^	1.0 × 10^−3^	6.67 × 10^−2^–6.67 × 10^−2^	6.67 × 10^−2^	6.67 × 10^−2^	1.0 × 10^−3^
Napo	2.34 × 10^−2^–4.22 × 10^−1^	2.34 × 10^−2^	3.77 × 10^−1^	1.64 × 10^−1^	6.67 × 10^−2^–1.20	6.67 × 10^−2^	1.07	4.66 × 10^−1^
Pastaza	2.34 × 10^−2^–4.22 × 10^−1^	9.38 × 10^−2^	3.60 × 10^−1^	1.39 × 10^−1^	6.67 × 10^−2^–1.20	2.67 × 10^−1^	1.03	3.97 × 10^−1^
Morona Santiago	2.34 × 10^−2^–5.25 × 10^−1^	2.34 × 10^−2^	3.95 × 10^−1^	1.47 × 10^−1^	2.34 × 10^−2^–1.49	6.67 × 10^−2^	1.12	4.18 × 10^−1^
Zamora Chinchipe	6.56 × 10^−2^–3.47 × 10^−1^	1.36 × 10^−1^	3.26 × 10^−1^	9.27 × 10^−2^	1.87 × 10^−1^–9.88 × 10^−1^	3.87 × 10^−1^	9.28 × 10^−1^	2.64 × 10^−1^

*n* = number of samples; min = minimum; max = maximum; p50 = percentile 50; p95 = percentile 95; S.D. = Standard Deviation.

**Table 4:** Probabilistic HQ and HI from exposure to Hg in groundwaters for both receptors. Values in bold exceed the safe exposure threshold.

Risk	Adults				Children			
	Min–Max	p50	p95	S.D.	Min–Max	p50	p95	S.D.
HQ_ingestion_	5.14 × 10^−3^–4.39 × 10^−1^	4.18 × 10^−2^	2.05 × 10^−1^	6.89 × 10^−2^	1.20 × 10^−2^–8.66 × 10^−1^	8.94 × 10^−2^	4.22 × 10^−1^	1.42 × 10^−1^
HQ_dermal_	8.55 × 10^−5^–7.42 × 10^−3^	7.02 × 10^−4^	3.58 × 10^−3^	1.17 × 10^−3^	2.65 × 10^−4^–2.71 × 10^−2^	2.54 × 10^−3^	1.19 × 10^−2^	4.13 × 10^−3^
HI	5.23 × 10^−3^–4.47 × 10^−1^	4.26 × 10^−2^	2.08 × 10^−1^	7.01 × 10^−2^	1.23 × 10^−2^–8.93 × 10^−1^	9.20 × 10^−2^	4.33 × 10^−1^	1.47 × 10^−1^

min = minimum; max = maximum; p50 = percentile 50; p95 = percentile 95; S.D. = Standard Deviation.

## Data Availability

The data presented in this study are available upon request from the corresponding author.
